# Molecular cytogenetic analysis and genetic counseling: a case report of eight 46,XX males and a literature review

**DOI:** 10.1186/s13039-019-0456-y

**Published:** 2019-11-04

**Authors:** Fagui Yue, Hongguo Zhang, Qi Xi, Yuting Jiang, Leilei Li, Ruizhi Liu, Ruixue Wang

**Affiliations:** 1 Center for Reproductive Medicine, Center for Prenatal Diagnosis, First Hospital, Jilin University, Changchun, 130021 China; 2 Jilin Engineering Research Center for Reproductive Medicine and Genetics, Jilin University, Changchun, 130021 China

**Keywords:** 46,XX male, Sex reversal, *SRY*, *DAX1* mutation

## Abstract

**Background:**

46,XX male syndrome is a rare disorder that usually causes infertility. This study was established to identify the genetic causes of this condition in a series of 46,XX males through the combined application of cytogenetic and molecular genetic techniques.

**Case presentation:**

We identified eight azoospermic 46,XX males who underwent infertility-related consultations at our center. They all presented normal male phenotypes. In seven of the eight 46,XX males (87.5%), translocation of the *SRY* gene to the terminal short arm of the X chromosome was clearly involved in their condition, which illustrated that this translocation is the main mechanism of 46,XX sex reversal, in line with previous reports. However, one patient presented a homozygous *DAX1* mutation (c.498G > A, p.R166R), which was not previously reported in *SRY*-negative XX males.

**Conclusions:**

We proposed that this synonymous *DAX1* mutation in case 8 might not be associated with the activation of the male sex-determining pathway, and the male phenotype in this case might be regulated by some unidentified genetic or environmental factors. Hence, the detection of genetic variations associated with sex reversal in critical sex-determining genes should be recommended for *SRY*-negative XX males. Only after comprehensive cytogenetic and molecular genetic analyses can genetic counseling be offered to 46,XX males.

## Background

46,XX male syndrome, also called *de la Chapelle* syndrome, is a genetic disorder encountered infrequently in a clinical context [1]. This syndrome is found in approximately 1 in 20,000–25,000 males, who exhibit a male phenotype but a 46,XX karyotype [2]. These patients have three main phenotypic manifestations: (1) classic XX males with infertility presenting normal male internal and external genitalia; (2) XX males with ambiguous genitalia presenting apparently external genital ambiguities at birth, such as hypospadias, micropenis, or hyperclitoridy; and (3) XX true hermaphrodites presenting internal or external genital ambiguities at birth [3–5].

Molecular findings on the presence of the sex-determining region Y (*SRY*) gene can be used to divide XX males into *SRY*-positive and -negative groups [6]. About 90% of XX males have the *SRY* gene, which plays a critical role in encoding the testis-determining factor (TDF) [7]. These *SRY*-positive XX individuals present normal genitalia and a male phenotype at birth [4]. However, in the *SRY*-negative 46,XX males, external genital ambiguities of different degrees are presented [5].

According to a literature review, several genes such as *SRY*, *SOX9*, *DAX1*, and *WNT4* are associated with sex reversal. Herein, we describe eight azoospermic 46,XX males presenting a normal male phenotype and masculinization. We also perform a literature review to investigate the correlation between *DAX1* mutation and XX males, aiming to explain the genetic cause of *SRY*-negative XX males.

## Case presentation

### Participants and clinical data

From 2015 to 2017, eight Chinese males underwent consultations for infertility at the Center for Reproductive Medicine and Center for Prenatal Diagnosis of the First Hospital of Jilin University because of no pregnancy resulting from regular unprotected coitus. The results of physical and routine clinical examinations were listed in Table 1. All patients were finally diagnosed with azoospermia based on routine semen examination [8]. The Ethics Committee of the First Hospital of Jilin University approved our study protocol (No. 2016–422) and all patients provided written informed consent to participate in this study.

**Table 1:** Summary of the physical and clinical examinations of our patients

Case	Age	Height (cm)	Weight (kg)	Testicle volume (mL)		Serum levels					Male signs	Diagnosis	ART follow-up
				Left	Right	FSH	LH	E2	PRL	T			
1	23	165	49	2	2	N.A.	N.A.	N.A.	N.A.	N.A.	Sparse beard	Azoospermia	AID
2	36	171	57	2	2	45	29.6	19.27	196	3.9	Normal	Azoospermia	AID
3	28	166	60	2	2	18.8	14.1	33.36	269	4.7	Normal	Azoospermia	AID
4	32	169	50	1	1	45.4	28.5	31.8	219	5.4	Sparse beard	Azoospermia	N.A.
5	26	175	80	3	5	21.2	14.3	22.63	147	6.5	Normal	Azoospermia	N.A.
6	30	170	60	4	4	62.83	27.12	17.9	316.6	9.19	Normal	Azoospermia	N.A.
7	23	165	56	3	3	40.05	22.11	13.6	316.6	2.22	Normal	Azoospermia	N.A.
8	27	168	51	10	10	6.6	9.6	33.14	485	3.6	Normal	Azoospermia	N.A.

The reference values were obtained from electrochemiluminescence immunoassays (ECLIA) using Roche Elecsys1010 (Roche Diagnostics, Mannheim, Germany) according to the manufacturer’s instructions. *N.A.* Not available, *ART* Assisted reproductive technology, *AID* Artificial insemination by donor sperm

FSH (follicle-stimulating hormone): 1.5–12.4 mIU/ml; LH(luteinizing hormone): 1.7–8.5 mIU/ml; E2(estradiol): 28–248 pg/ml; PRL(prolactin): 86–258 uIU/ml; T(testosterone): 9.9–27.8 nmol/l

## Methods

### Karyotype analysis

Conventional G-banding techniques were applied on the cultured peripheral blood cells for chromosomal karyotyping. We described all of the chromosomal karyotypes according to the ISCN 2016 nomenclature [9].

### AZF microdeletion analysis

Microdeletions in the AZF region were detected using polymerase chain reaction (PCR), as previously described in accordance with the recommendations of the European Academy of Andrology and the European Molecular Genetics Quality Network. Specific sequence-tagged sites (STSs) were mapped in the AZF region, including SY84 and SY86 for AZFa; SY27, SY134, and SY143 for AZFb; SY157, SY254, and SY255 for AZFc; and SY152 for AZFd [10].

### Fluorescence in situ hybridization analysis (FISH)

FISH specific for the Y chromosome was performed on metaphase slides for the patients to further confirm the existence of *SRY* through the standard operating protocol (Cytocell Technologies, Cambridge, UK). The detecting probes were as follows: red-labeled sex-determining region Y (*SRY*) probe with two nonoverlapping probes, green-labeled probe for a heterochromatic region (DYZ1) in Yq12, and blue-labeled probe for the X centromere (DXZ1).

### Sanger sequencing

Some genes had been shown to be critically involved in sex differentiation, such as *SOX9*, *DAX1*, and *WNT4* [11, 12]. Sequencing was performed to detect mutations in these genes on an ABI 3730xl DNA analyzer (Applied Biosystems) by BGI (Beijing, China) for the *SRY*-negative patients [13].

## Results

**Fig. 1:**
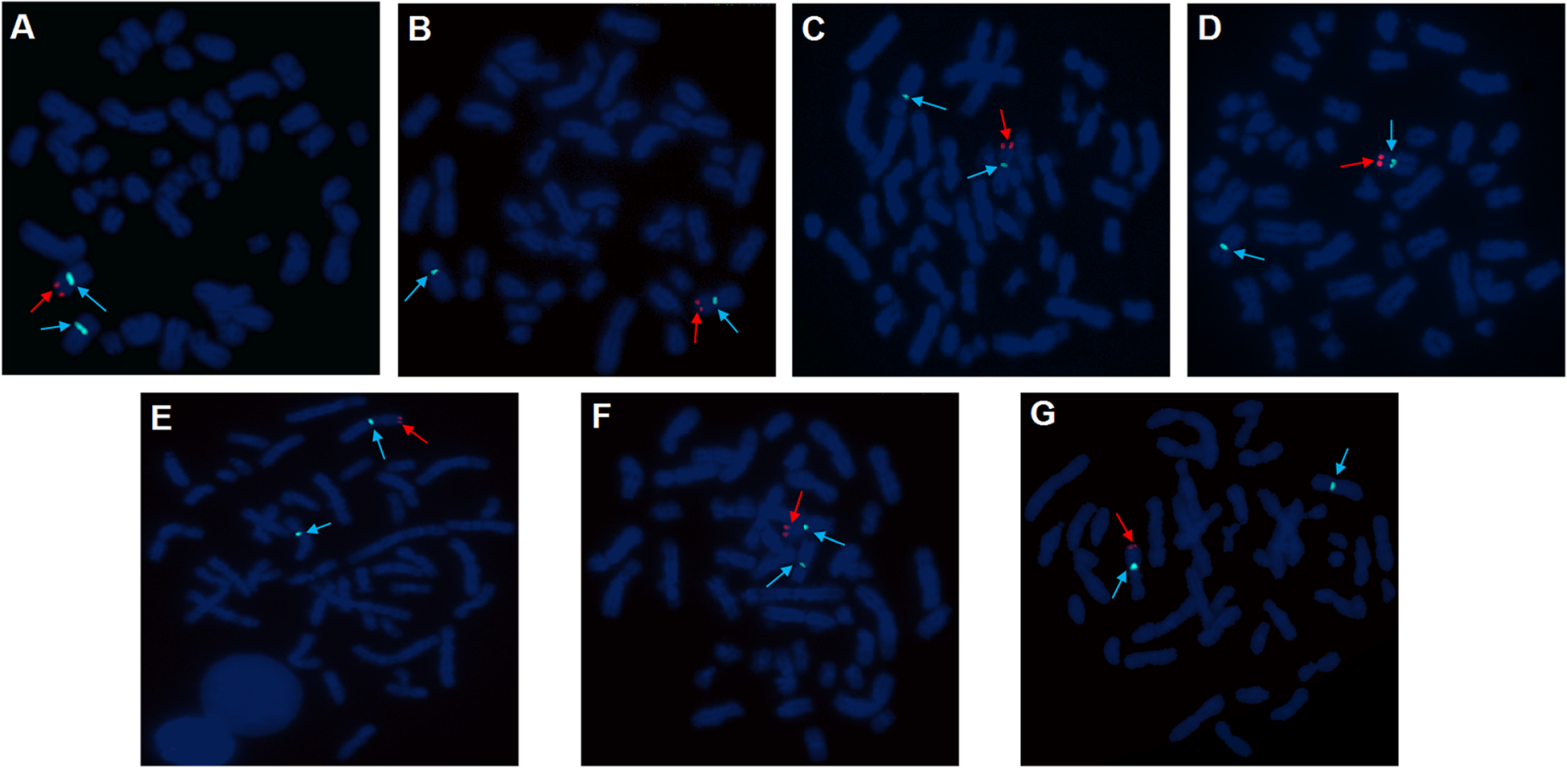
FISH demonstrated that the SRY region was located on the distal tip of the short arm of the chromosome X. **a**:case 1. **b**:case 2. **c**: case 3. **d**: case 4. **e**: case 5. **f**: case 6. **g**: case 7. Red arrows indicated SRY signal (red), and blue arrows indicated X centromere (blue)

**Fig. 2:**
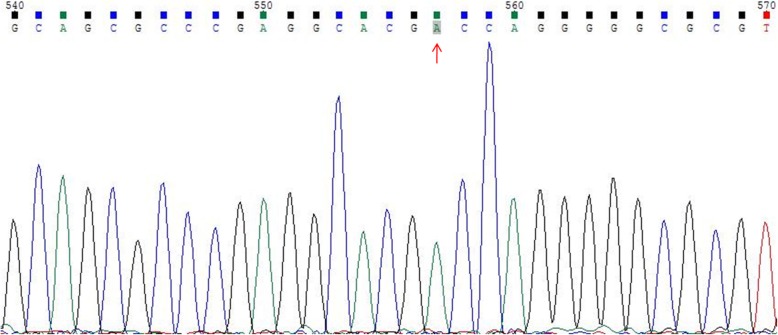
Sanger sequencing analysis of DAX1 gene for case 8: a synonymous mutation of DAX1 (c.498G > A, p.R166R)

The results of cytogenetic G-banding and AZF microdeletion analyses were listed in Table 2. All of the 46,XX males reported here exhibited AZFa+b + c microdeletion. FISH confirmed the presence of a translocated *SRY* region located on the distal tip of the short arm of the X chromosome in seven patients (cases 1–7, Fig. 1). For case 8, the PCR analysis demonstrated the absence of the *SRY* gene; as such, Sanger sequencing was performed on three key genes (*SOX9*, *WNT4*, and *DAX1*) associated with sex reversal. No mutations were discovered in the coding regions of *SOX9* and *WNT4*. However, a homozygous variant in exon 1 of *DAX1* (c.498G > A, p.R166R) was detected (Fig. 2), which was not previously reported in *SRY*-negative 46,XX males. Only cases 1 to 3 chose artificial insemination with donor sperm, according to the assisted reproductive technology follow-up outcomes.

**Table 2:** Sequence-tagged site deletions and chromosomal analysis

Case	AZFa		AZFb			AZFc				Karyotype analysis
	SY86	SY84	SY127	SY134	SY143	SY152	SY254	SY255	SY157	
1	–	–	–	–	–	–	–	–	–	46,XX
2	–	–	–	–	–	–	–	–	–	46,XX
3	–	–	–	–	–	–	–	–	–	46,XX
4	–	–	–	–	–	–	–	–	–	46,XX
5	–	–	–	–	–	–	–	–	–	46,XX
6	–	–	–	–	–	N.A.	–	–	N.A.	46,XX
7	–	–	–	–	–	–	–	–	N.A.	46,XX
8	–	–	–	–	–	–	–	–	–	46,XX

*AZF* Azoospermic factor, *STS* Sequence-tagged site; −, deletion of specific STS, *N.A.* Not available

## Discussion and conclusions

In this paper, we reported eight azoospermic cases of 46,XX males with normal male genitalia and no apparent abnormalities. All subjects presented 46,XX karyotypes and AZFa+b + c microdeletion. Among them, seven cases were *SRY*-positive (87.5%), with the *SRY* gene being translocated to the short end of the X chromosome, while the eighth case was *SRY*-negative (12.5%) with a synonymous mutation of *DAX1* (c.498G > A, p.R166R).

The first case of 46,XX male syndrome was reported in 1964 [1]. The clinical manifestations are mainly characterized by a normal phenotype in newborns, but delayed puberty, gynecomastia, or infertility in adolescents. In addition, hypospadias, cryptorchidism, and severely ambiguous genitalia can also be observed [14]. The majority of XX males were found to be *SRY*-positive, presenting sterility with normal male genitalia and *SRY* translocation to the X chromosome or autosomes. However, reports on *SRY*-negative XX males have been limited, with these cases usually presenting infertility with immature/ambiguous to normal genitalia, incomplete testicular development or ovotestis, and varying degrees of masculinization [15].

The mechanism of 46,XX male syndrome in *SRY*-positive cases could be summarized as involving cross-over errors in the pseudoautosomal regions of the sex chromosome during paternal meiosis [16]. However, the mechanism in *SRY*-negative 46,XX males has remained unclear. Several possibilities were proposed to explain these cases: mosaicism for *SRY* in hidden gonads, the inhibition of male pathways resulting from mutations of autosomal or X-linked genes, and mutations of other sex-determining genes downstream of *SRY* [14, 15].

The sex of individuals is known to be determined by the *SRY* gene in most mammals, but the existence of *SRY*-negative males demonstrates the involvement of other genes in determining maleness in the absence of *SRY*. Mutations of some critical genes, such as *SOX9*, *DAX1*, and *WNT4*, have been proven to be associated with sex reversal [11, 12]. *DAX1* (dosage-sensitive sex reversal adrenal hypoplasia congenital critical region of the X chromosome gene 1), also called *NR0B1*, is located on chromosome Xp21.3-p21.2 (ΟMIM#300473). It contains two exons and one intron, which encode an orphan nuclear receptor. *DAX1* is widely expressed in the adrenals, hypothalamus, pituitary, and testis, playing critical roles in testicular and ovarian development. Mutations of *DAX-1* are usually associated with primary adrenal insufficiency or congenital adrenal hypoplasia (CAH) and hypogonadotropic hypogonadism (MIM #300200) [17, 18]. *DAX1* was initially recognized as a dosage-sensitive ovarian-determining gene. An increased number of copies of *DAX1* could lead to high expression of its encoded proteins, which would result in sex reversal [11]. Further research revealed that *DAX1* was actually necessary for testis differentiation and spermatogenesis [19, 20]. Zenteno et al. [21] assumed that *SRY*-negative XX males with normal genitalia were homozygous for deletions or loss-of-function mutations in dosage-sensitive sex reversal. In addition, Domenice et al. [12] proposed that the loss of function of the *DAX1* gene might prevent its repressive effect on masculinizing genes and thus determine testicular development in XX individuals, which probably explained the presence of 46,XX sex reversal. In addition, Dangle et al. [22] reported a 46,XX *SRY-*negative case with a heterozygous deletion encompassing *DAX1*.

In case 8 presented here, Sanger sequencing of the coding regions of the *DAX1* gene showed a synonymous mutation of this gene (c.498G > A, p.R166R), which was also described in other reports. For example, Mou et al. [23] reported a series of patients with secretory azoospermia and fertility who presented synonymous mutation (c.498 G > A) in *DAX1*. In addition, Achermann et al. [24] described the same *DAX1* mutation in patients with hypogonadotropic hypogonadism or pubertal delay. Moreover, patients with CAH could also present *DAX1* mutation (c.498 G > A), in those with disease onset in either infancy or adulthood [25, 26]. Xu et al. [27] reported a 3-year-old boy who was diagnosed with X-linked CAH, with three novel mutations detected in *DAX1*: a missense mutation (c.376G > A, p.Val126Met), a synonymous mutation (c.498G > A, p.Arg166Arg), and a nonsense mutation (c.1225C > T, p.Gln409X). Currently, the mechanisms triggering testis development in SRY-negative 46,XX males remain unknown. Overexpression of the *DAX1* gene could cause female-to-male sex reversal [28], but this was not analyzed in case 8, as we failed to investigate probably hidden gonadal mosaicism for *SRY* or mutations in autosomes, or other functional mutations of unknown sex-determining genes (e.g., *SF1*, *RSPO1*, *SOX3*, *SOX10*, *ROCK1*, and *DMRT*) [14]. Considering the pathogenicity of the polymorphism as recorded in the ClinVar database, the potential risk of CAH in case 8 should be considered, besides the sex reversal. Despite the *DAX1* mutation detected in case 8 not previously being reported in *SRY*-negative 46,XX males, the potential association between *DAX1* mutation and *SRY*-negative 46,XX males still requires further investigation. We also speculated that other unidentified genetic or environmental factors might play critical roles in regulating sex determination and gonad sex differentiation.

The *SOX9* and *WNT4* genes were also sequenced in case 8. The *SRY*-box 9 (*SOX9*) gene, located in 22q13, is a widely expressed transcription factor involved in male sex determination. Normal expression of *SOX9* was found to be associated with testicular differentiation. However, its overexpression or duplication might lead to 46,XX male sex reversal and testicular differentiation in the absence of *SRY* [29, 30]. With regard to *WNT4*, located in 1p36.12, it has also been shown to play a critical role in the development of the reproductive system as a candidate ovary-determining gene or antitestis gene [31]. Moreover, it has been reported that loss-of-function mutation in *WNT4* could result in partial XX male sex reversal [32]. Given their lack of clear causative mutations in this study, *SOX9* and *WNT4* might not be critical factors in *SRY*-negative XX males.

In other words, irrespective of the status as *SRY*-positive or -negative, 46,XX males would always present infertility due to the absence of the AZFa, AZFb, and AZFc regions located on chromosome Yq11, which are involved in regulating normal spermatogenesis [14].

In the present study, eight cases of 46,XX male syndrome were identified based on cytogenetic and molecular genetic analyses. One *SRY*-negative XX male carried a homozygous p.R166R synonymous mutation in *DAX1*, while the other seven *SRY*-positive 46,XX individuals had *SRY* translocated to the terminal of the X chromosome. Our findings enrich the understanding of the genotype–phenotype correlation in 46,XX males, especially in patients with *SRY*-negative female-to-male sex reversal. The combined application of chromosomal analysis, AZF microdeletion evaluation, *SRY* detection, and sequencing of key sex-determining genes should be recommended for these patients.

## Data Availability

The data and material used or analysed during the current study are available from the corresponding author on reasonable request.

## Abbreviations

AZF: Azoospermia factor
FISH: Fluorescent in situ hybridization
ISCN 2016: International System for Human Cytogenetic Nomenclature 2016
*SRY*: Sex-determining region Y
STS: Specific sequence-tagged sites
